# The emerging roles of exosomes in leukemogeneis

**DOI:** 10.18632/oncotarget.9333

**Published:** 2016-05-12

**Authors:** Jianbiao Zhou, Sam Wang, Kangyun Sun, Wee-Joo Chng

**Affiliations:** ^1^ Cancer Science Institute of Singapore, National University of Singapore, Centre for Translational Medicine, Singapore, Republic of Singapore; ^2^ Department of Medicine, Yong Loo Lin School of Medicine, National University of Singapore, Singapore, Republic of Singapore; ^3^ Faculty of Arts and Sciences, University of Toronto, Toronto, ON, Canada; ^4^ Suzhou Municipal Hospital, Affiliate of Nanjing Medical University, Suzhou, Jiangsu Province, PR China; ^5^ Department of Hematology-Oncology, National University Cancer Institute of Singapore (NCIS), The National University Health System (NUHS), Singapore, Republic of Singapore

**Keywords:** exosome, leukemia, immunotherapy, biomarker, miRNA

## Abstract

Communication between leukemia cells and their environment is essential for the development and progression of leukemia. Exosomes are microvesicles secreted by many types of cells that contain protein and RNA and mediate intercellular communication. The involvement of exosomes has been demonstrated in the crosstalk between leukemic cells, stromal cells and endothelial cells, consequently promoting the survival of leukemic cells, protection of leukemic cells from the cytotoxic effects of chemotherapeutic drugs, angiogenesis and cell migration. At the same time, exosomes can be used for the detection and monitoring of leukemia, with some advantage over current methods of detection and surveillance. As they are involved in immune response towards leukemic cells, exosomes can also potentially be exploited to augment immunotherapy in leukemia. In this review, we first describe the general characteristics of exosomes and biogenesis of exosomes. We then highlight the emerging role of exosomes in different types of leukemia. Finally, the clinical value of exosomes as biomarkers, *in vivo* drug carriers and novel exosome-based immunotherapy are discussed.

## INTRODUCTION

Leukemia is a group of malignant diseases originating from blood or bone marrow cells, including chronic myeloid leukemia (CML), acute myeloid leukemia (AML), chronic lymphoblastic leukemia (CLL) and acute lymphoblastic leukemia (ALL). In the US alone, there are more than 50, 000 new cases diagnosed with leukemia per year. The prognosis of leukemia patients diverges greatly, largely depending on the type of leukemia and the age of the patient. Overall, leukemia remains a significant challenge for hematologists and oncologists as it ranks as the 5th and 6th most common cancer death in men and women, respectively (Facts and Statistics 2015, Leukemia and Lymphoma Society). A better understanding of leukemogensis will lay the foundation for the development of novel anti-leukemic therapies.

**Figure 1:**
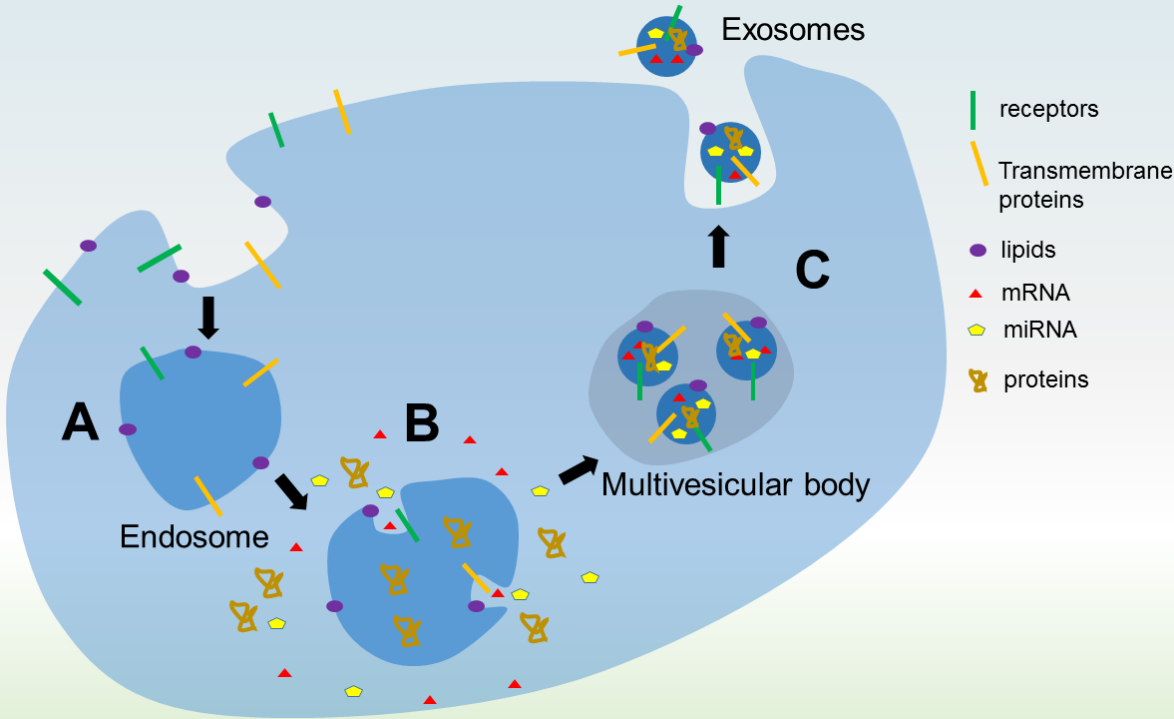
Biogenesis of exosomes **A.** An endosome forms from invagination of the plasma membrane to surround macromolecules or other cargos within the extracellular environment. Proteins on the plasma membrane are found on the endosomal membrane. **B.** Invagination of the endosomal membrane forms a multivesicular body (MVB). Intracellular proteins and RNA are packaged into the microvesicles in the MVB. **C.** Fusion of the MVB with the plasma membrane releases the microvesicles as exosomes into the extracellular milieu in an exocytic process.

Exosomes are found in most, if not all, biological fluids including urine, blood, ascites and cerebrospinal fluid [1, 2]. These nanoparticles contain bilayer-lipid membrane and have cup-like shape with diameters between 30 and 150 nm [3]. Ectosomes are another type of extracellular vesicles typically measured as 150 - 1000 nm [4]. Exosomes are continuously secreted by cell through endocytosis in multi-vesicular bodies (MVBs) and released by the fusion of MVBs with the plasma membrane [3, 5, 6]. The endosomal sorting complex required for transport (ESCRT) complex consists of an array of membrane-associated proteins and coordinates the process of MVBs generation [7]. The identification of some ESCRT proteins, such Alix and Tsg101, in exosomes by proteomic analysis supports a role for ESCRT complex in exosome biogenesis [8]. The step wise biogenesis of exosomes is illustrated in Figure 1. Exosomes characteristically contain tubulin, actin, actin-binding proteins, annexins, Rab proteins, MHC class I, MHC class II, heat shock proteins and the tetraspanins CD9, CD63, CD81 and CD82, along with small RNA and messenger RNA found in the cytoplasm of their cell of origin [8-11]. First discovered in 1983 from sheep reticulocytes, these microvesicles were initially believed to be a way by which cells disposed of unnecessary membrane proteins during the process of maturation [2]. Since then, considerable work has been done on exosomes and they have been shown to mediate a variety of physiological and pathological processes *via* their interaction with proximal or distant target cells [12]. The functions of exosomes have been demonstrated to play important roles in the survival and proliferation of cancer cells and metastasis [13-15]. On the other hand, exosomes have also been found to be potentially useful in improving cancer treatment, detection and prognosis [16, 17]. This review will focus on recent developments in exosome research in relation to leukemia.

## IMMUNE ESCAPE OF LEUKEMIC CELLS

**Figure 2:**
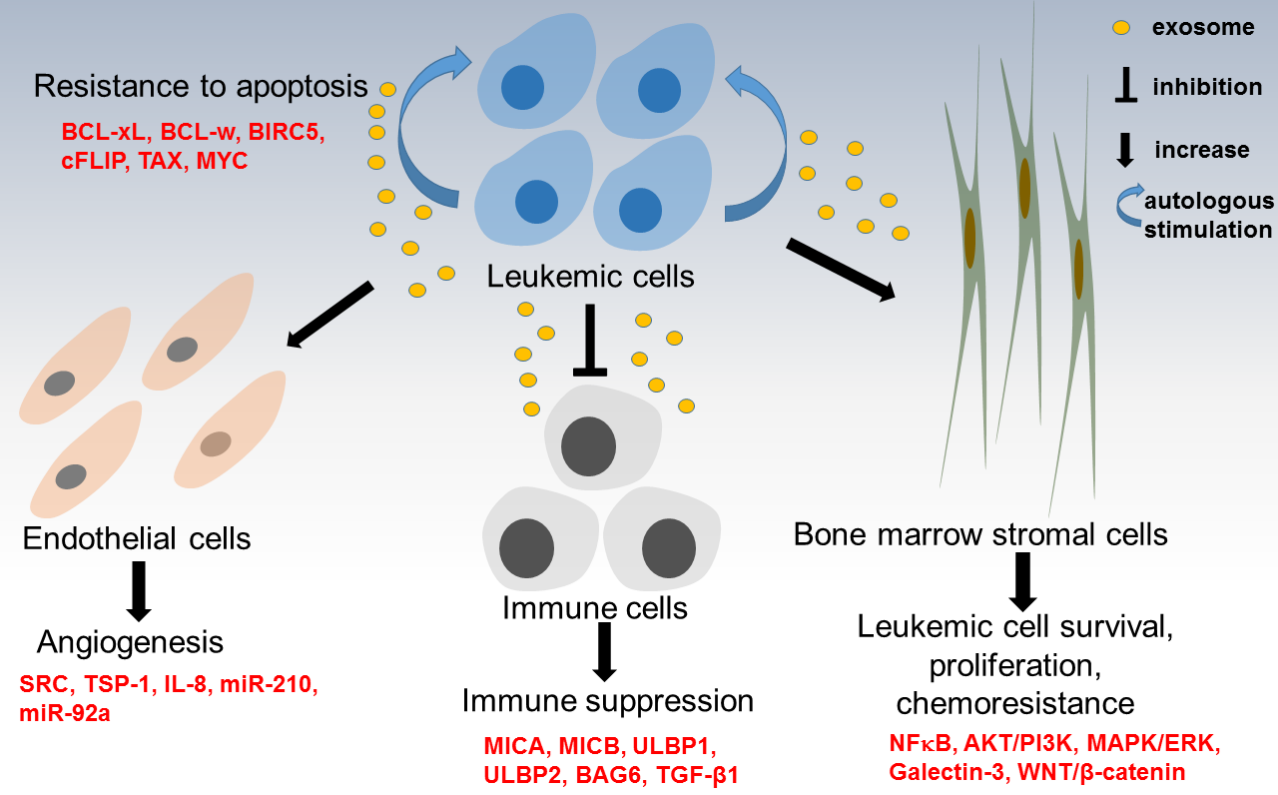
Summary of role of exosomes in leukemogenesis Leukemia-derived exosomes can activate bone marrow stromal cells (BMSCs) to promote leukemia cell survival, proliferation and resistance to chemotherapy. Leukemia-derived exosomes impair the host immune system to create an immunosuppressive state, facilitating immuoescape of leukemia cells. Leukemia-derived exosomes transfer pro-angiogenic molecules into endothelial cells to enhance angiogenesis and provide a favourable microenvironment for leukemia cells themselves. Moreover, leukemia-derived exosomes can help leukemia cells develop resistance to apoptosis through transferring pro-survival BCL-2 from apoptosis-resistant AML blasts to apoptosis-sensitive AML cells.

Cancer cells can evade host immune surveillance, a well-known phenomenon called as immune evasion or immune escape, which is also a hallmark of cancer. Cancer cells exploit several immunological mechanisms, such as down-regulation of target antigens, targeting regulatory T-cell functions, or secretion of immune suppressive mediators [18]. Transforming growth factor beta 1 (TGF-β1) is a major secreted cytokine that inhibits helper T-cells and cytotoxic T-cells [19, 20]. It has been recently demonstrated that exosomes released by leukemic cells exert an immunosuppressive effect that helps them evade immune response. Study from Szczepanski and coworkers demonstrated that sera from AML patients contained higher level of exosomes and distinct molecule profiles in exosomes as compared to that of sera from healthy controls [21]. Exosomes isolated from the sera of AML patients contain membrane-associated TGF-β1, which reduces the ability of natural killer (NK) cells to kill leukemic cells by reducing NKG2D expression and activating the SMAD pathway [8]. In a follow-up study, the level of exosomal TGF-β1 has been shown to correlate to response to chemotherapy in AML patients [22]. Jurkat and Raji leukemia/lymphoma cells increase their release of exosomes that express the NKG2D ligands MICA, MICB, ULBP1 and ULBP2 on their membranes. These ligands bind to NKG2D and impede the NKG2D ligand-receptor pathway in NK cells, thereby reducing their capacity to kill leukemic cells [9]. Exosomal BAG6, the ligand for the receptor NKp30 expressed on NK cells, is essential for NK cells to kill cancer cells and is believed to be down-regulated or absent in CLL patients as suggested by the immune suppression observed in CLL patients [10]. In addition, TGF-β1 has also been found to be enriched in CML-exosomes and treatment with TGF-β1 receptor inhibitor (SB) significantly reduces exosome-stimulated cell proliferation and colony formation of CML cells. Furthermore, Exosomal TGFβ1 also has been shown to be critical in the formation of tumour-promoting stroma, down-regulates NKG2D expression and inhibits CTL response in solid tumor models [23-28]. Taken together, these evidence suggest exosome-mediated NK cell dysfunction compromise the immune surveillance to eliminate leukemic cells in various hematologic malignancies (Figure 2). TGF-β1 plays a pivotal role in leukemic exosome-mediated immune escape.

## LEUKEMIC CELL SURVIVAL AND PROLIFERATION

Leukemic cells release exosomes that are internalized by nearby cells [29, 30]. Through this process, cancer cells are able to transfer proteins and RNA to surrounding cells [29, 30]. Moreover, exosomes are able to travel in extracellular space and deliver exosomal cargo into distant cells. Exosomes released by AML cells enrich mRNA transcripts of genes important to the development of leukemia including GATA1, FOX3, SHIP1, ID1, E2F1, CEBP-α, CEBP-β, Myc and MEF2C [29]. Exosomes secreted by LAMA84 CML cells increase IL-8 mRNA and protein levels in HS5 bone marrow stromal cells (BMSCs), which, in turn, promotes adherence of LAMA84 cells to a HS5 monolayer [31], a known promoter of cell survival [32, 33]. LAMA84 cell-derived exosomes also directly promote the survival of LAMA84 cells by lowering expression of the pro-apoptotic genes BAD, BAX and PUMA, elevating expression of anti-apoptotic genes BCL-xL, BCL-w, and BIRC5. Moreover, these secreted exosomes also increases NF-kB and TGF-β1 levels and activates the PI3/AKT and MAPK/ERK signaling pathways [34]. Infection by human T-lymphotropic virus type 1 (HTLV1) is one of the main causes of adult T-cell leukemia [16]. HTLV1 infected T cells release exosomes that contain viral Tax protein and Tax mRNA transcripts. In addition, they enhance cell survival in murine and human T-cell cell lines by increasing levels of phosphorylated AKT and Rb and protect Jurkat cells from Fas-mediated apoptosis by increasing cFLIP and NF-kB activity, demonstrating an important role in the development of leukemia played by exosomes [35] (Figure 2).

## ANGIOGENESIS IN LEUKEMIA

Angiogenesis, the formation of new blood vessels, is indispensable for tumor progression, especially for tumors with size more than 1 - 2 mm^3^ [36, 37]. Exosome has been increasingly recognized as a new mediator for this critical step. Exosomes from CML cells induce the formation of a tube-like structure in human umbilical vein endothelial cells (HUVECs) by transferring miR-92a and activating SRC signaling [38, 39]. Activation of the Src kinase family is required for oncogenic signaling by BCR-ABL, a characteristic feature of CML [40]. Exosomes isolated from K562 cell culture supernatant induces phosphorylation of Src protein and activates downstream Src kinase signaling pathways [38]. Importantly, this activation process can be blocked by Dasatinib, a BCR-ABL and Src dual inhibitor [38]. In *in vitro* experiment, K562 exosomes stimulate angiotube formation and increases total cumulative tube length 2-fold compared with control. MiRNAs are a group of evolutionarily conserved, single-stranded, 22 nucleotides non-coding small RNAs. Many miRNAs have been shown to promote or inhibit tumor angiogenesis though post-transcriptional regulation of pro-angiogenic or anti-angiogenic molecules [41]. One prominent example, oncogenic miR-17-92 family, is known to stimulate angiogenesis in the adjacent tumor endothelium by direct repression of the secreted, antiangiogenic molecules thrombospondin-1 (TSP-1) and connective tissue growth factor (CTGF) [42]. Interestingly, miRNA-92a, a member of miR-17-92 cluster, is found presence in exosomes released by K562 cells and these K562 exosomes down-regulate integrin a5, a target gene of miR-92a, in HUVECs. Enforced expression of miRNA-92a significantly enhances cell migration and tube formation of HUVECs [39].

Consistent with current knowledge of hypoxia being a key driver of angiogenesis, CML exosomes secreted in hypoxic conditions are better able to increase tube formation in HUVECs compared to exosomes from cells cultured in normoxia, attributable to heightened exosomal levels of miR-210 which down-regulated EFNA3, an inhibitor of angiogenesis [43]. Exosomes from LAMA84 cells increase IL-8 expression and MAPK phosphorylation in HUVECs, which causes a similar angiogenic effect [44]. One study investigated the angiogenic role of extracellular vesicles (EVs) produced by NB4 cells, a AML-M3 subtype leukemia. In this study, PML-RARa transcript has been detected in NB4 EVs and taken up by endothelial cells, resulting in these endothelial cells being more tissue factor-positive and procoagulant [45]. In summary, these results suggest that leukemia cells release exosomes containing pro-angiogenic molecules, which are transferred into endothelial cells to create a microenvironment favorable to survival and proliferation of leukemia cells themselves (Figure 2).

## CHEMORESISTANCE

Bone marrow stromal cells (BMSCs) are an important component of bone marrow niche and they may differentiate into bone, cartilage, adipocytes and the hematopoiesis-supporting stroma. Many studies indicate that BMSCs enhance survival of leukemia cells and hinder apoptosis of these cells resulted from chemotherapy [46-49]. However, the exact mechanisms of this protection from chemotherapeutic attack are not fully understood. Emerging evidence suggests that both the exosomes released from BMSCs and the leukemia cells themselves help the leukemia cells to resist chemotherapy. Leukemic exosomes increase the levels of proteins involved in chemoresistance in BMSCs, indicating a role in communication between the cell types and in the transfer of substances relevant to chemoresistance. Exosomes from both normal BMSCs and BMSCs from AML patients reduce the cytotoxic effects of the nucleoside analogue cytarabine on MOLM-14 cells with FLT3-ITD (internal tandem duplication) mutation, and only AML-BMSC derived exosomes provided such protection from AC220 (a FLT3 kinase inhibitor) treatment [50]. The protection might be associated with elevated level of miRNA-155, miRNA-375, cytokine epidermal growth factor (EGF) and TGF-β1 [50]. However, a direct causal effect of these factors on the chemoresistance has not been interrogated. Nevertheless, these data imply a few novel approaches to increase chemosensitivity on AML blasts by either blocking exosome-leukemia cell communication, or inhibiting exosome production.

Galectin-3 is a member of the lectin family, as well as a member of the beta-galactoside-binding protein family [51]. Galectin-3 functions as a modulator of cell-cell adhesion, cell-matrix interactions, tumor angiogenesis and metastasis, immune response and apoptosis [52, 53]. Although contradictory results being reported, in general, the expression of galectin-3 is significantly higher in multiple tumors and is an indicator of metastatic potential [54]. Moreover, galectin-3 can be released by cancer cells into extracellular environment, thus the level of galectin-3 in the serum of many types of cancer patients are higher than healthy controls [55]. Together, these data suggest that increased expression of cellular galectin-3 and elevated concentration of galectin-3 in circulating system may contribute to tumor progression and metastasis. Several studies unveiled that increased galectin-3 facilitates leukemia cells escape from apoptotic stimuli through activation of Wnt/β-Catenin signaling pathway [56, 57]. High level of galectin-3 expression is an independent unfavorable prognostic factor for patients with AML, a protein associated with chemoresistance [56-59]. When B lineage ALL cells in co-culture with BMSCs, the stromal cells provide the ALL cells with galectin-3. The strong induction of galectin-3 coincides with observations that ALL cells in contact with OP9 cells are resistant to nilotinib, a BCR-ABL kinase inhibitor, and vincristine [60]. Galectin-3 is enriched in OP9 exosomes, but not ALL exosomes, and exosomal galectin-3 can be internalized by ALL cells, and activates NFkB pathway, which is often linked to anti-apoptosis and drug resistance [60] (Figure 2).

## DIAGNOSIS AND PROGNOSIS

Although useful in detecting disease, current methods for the diagnosis of leukemia have their limitations. Flow cytometry depends on the presence of circulating blasts and is inept at detecting leukemic cells that persist in the bone marrow in cases where circulating blasts have been eliminated by chemotherapy. Another currently employed method of detection is bone marrow aspiration, which is invasive and carries the risk of false negatives as the sample taken is not representative of all bone marrow tissue [61]. Due to their ubiquity in bodily fluids and cargo of proteins and RNA representative of their cells of origin, exosomes may contain biomarkers for cancer [62]. In other types of cancer such as pancreatic cancer, exosomal biomarker, Glypican-1, has already demonstrated ability to distinguish between healthy controls and advanced cancer patients with 100% accuracy [63]. Exosomal miRNA can be quantified from exosomes from just 20 μL of serum [62], allowing for a non-invasive method of detection of leukemia. Microvesicles are found in higher concentrations in the sera of AML, CML and CLL patients and abundantly express surface proteins unique to their cell of origin, which is rarely observed from serum microvesicles of normal controls [64]. AML biomarkers NPM1, FLT3, CXCR4, MMP9 and IGF-IR are also present in AML cell derived exosomes, along with mRNAs involved in leukemia development [29]. Isolation and analysis of serum exosomes can therefore shed light on the presence and progression of leukemia. In the case of CLL, the concentration of microvesicles and microvesicles expressing CD19 was directly proportional to leukemia advancement [64]. Recent research into the use of serum exosomes for diagnosis and prognosis of leukemia has produced promising results, with findings that exosomal TGF-β1 levels and relative levels of the three TGF-β1 forms (TGF-β1 pro-peptide, latency-associated peptide (LAP), and mature TGF-β1) were distinct in AML patients in different stages of chemotherapy [22], and that a grading system based on exosomal levels of miR-150, miR-155, miR-221 and miR-1246 was able to effectively distinguish AML cell xenografted mice from control mice [61]. Given the advantage that exosomal cargos, especially miRNAs, are protected from degradation by ribonuclease in the extracellular space, this stability makes exosomes as suitable mines for hunting reproducible and consistent biomarkers.

## TREATMENT OF LEUKEMIA

The relevance of exosomes in leukemia treatment comes mainly from its potential use in the loading of dendritic cells (DCs) [65], as drug delivery system or delivery vector for siRNA [66] or miRNA [67]. Other therapeutic strategies are to target tumor exosomes by either inhibition of their biogenesis [68-70] or extracorporeal hemofiltration of exosomes [71]. Suppression of exosome production by indomethacin has been shown to increase chemotherapeutic sensitivities of B-cell lymphoma [70]. DCs present cancer cell antigens on MHC class I or class II to T cells to bring about cytotoxicity towards cancer cells [72]. DC mediated immunotherapy combined with chemotherapy produces the longest survival times in DBA2 mice bearing murine L1210 lymphocytic leukemia tumor [73]. In studies involving both human and murine leukemia cells, loading of DCs with leukemic exosomes resulted in T cells having greater ability to kill leukemic cells compared to DCs alone or DCs loaded using leukemic cell lysate [74, 75]. Exposure to leukemic exosomes before the introduction of leukemic cells also increased cytotoxic activity towards leukemic cells and impeded tumor growth [76]. Antigens can be artificially introduced to exosomes. For example, gp350, an Epstein-Barr virus (EBV) protein, is expressed on the surfaces of exosomes released by HEK293 cells transfected with gp350. Gp350 binds to CD19 found on B cells and specifically targets them, delivering proteins such as CD154 that activated T cells and caused the B cells to be killed more effectively [77]. Meanwhile, exosomes released by DCs have been shown to induce immune response [78], the effectiveness of which can be increased by pulsing DC-derived exosomes with leukemic antigens [43].

Another potential use of exosomes in leukemia treatment is in the treatment of graft *versus* host disease (GvHD). GvHD can develop following hematopoietic stem cell or bone marrow transplantation and involves attack on the recipient's tissues by the donor's immune cells. Injections of mesenchymal stem cells (MSCs) are more effective at treating GvHD than conventional immunosuppressants [79]. It is possible that exosomes play a role the immunosuppressive effects of MSCs, as injections of MSC-derived exosomes were effective at treating GvHD and were able to reduce secretion of the cytokines IL-1β, TNF-α and IFN-γ by peripheral blood mononuclear cells [62]. It should also be noted that exosomes could replace liposomes as a vector in delivery of chemotherapeutic drugs. Exosomal delivery system would have the added benefits of slower removal from the bloodstream leading to increased effectiveness of delivery, as well as higher specificity for target cells [80-82].

## CONCLUSIONS

**Figure 3:**
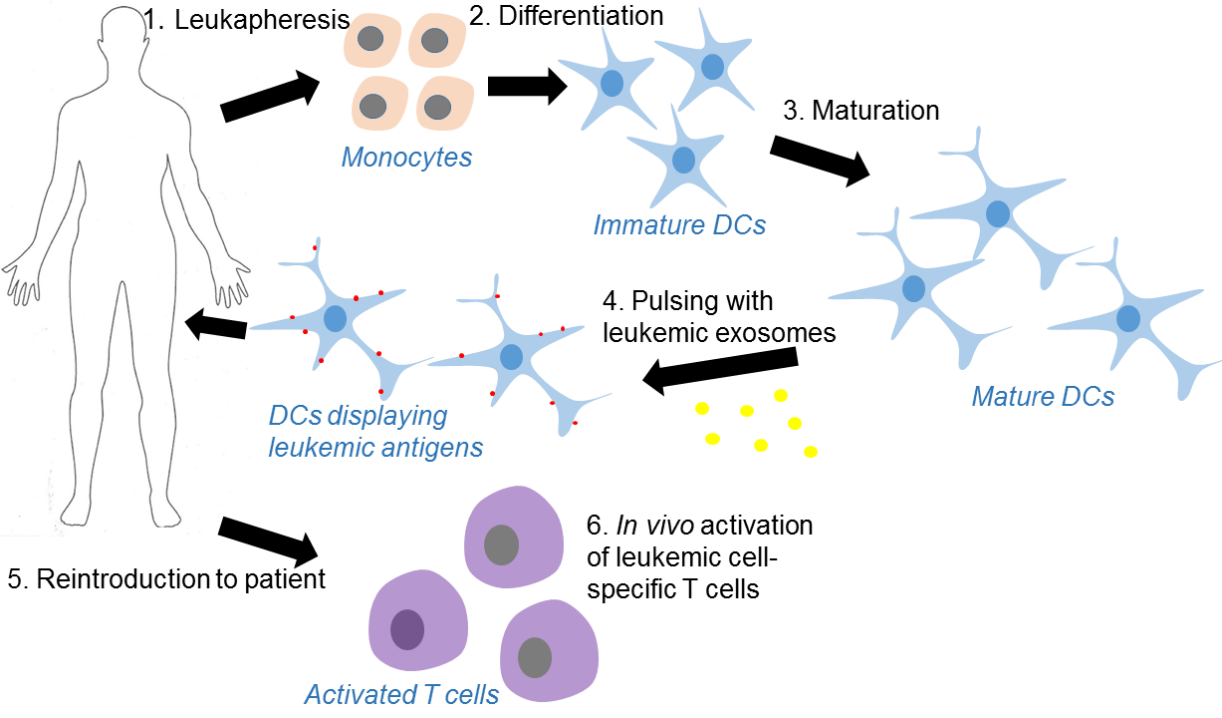
Process of dendritic cell-based leukemia immunotherapy using leukemia cell-derived exosomes as the source of antigens **1.** Monocytes are harvested from a leukapheresis, **2.** then differentiated *in vitro* under GMP-conditions into immature dendritic cells (DCs). **3.** The principal cytokines used for the development and maturation of DCs are GM-CSF and IL-4. **4.** Mature DCs are pulsed with leuekmia exosomes which contain leukemia patient specific antigens. **5.** DCs displaying leukemia antigens are infused into patients. **6.** DC-derived exosomes carry intact MHC class I or II-peptide complexes can induce target specific immune response in a T-cell-dependent fashion through either a direct or indirect mechanisms.

Exosomes are endosome-derived, membraneous nano-size microvesicles actively secreted by wide range of normal cells, as well as malignant cells. Leukemic cell derived exosomes contain cargoes such as miRNAs, proteins, mRNA, etc, which might represent a snapshot of the disease states of a leukemia patients. These cargoes, some of which are oncogenic, can be transferred into neighbor cells or distant cells and impact the behaviors of these receipt cells. Thus, exosome-mediated cell-cell communications could play potential important roles during leukemia development. Many studies provide evidence that exosomes are involved in survival and proliferation of leukemic cells, resistance to apoptosis and chemotherapeutic drugs, angiogenesis and migration. Notably, there is great interest in identification of exosomal biomarkers from blood samples for both diagnosis and stratification of leukemia patients. Although some pioneer studies reported some potential biomarkers, such as exosomal miR-126 in CLL, exosomal IGF-1R and TGF-β1 in AML, the real clinical utilities of these biomarkers are waiting for validation in large, perspective clinical trials. The potential use of exosomes to deliver tumor-derived antigens to elicit an immune response to specific cancer antigens has been confirmed in several phase I and II clinical trials in melanoma, non-small cell lung cancer and colorectal cancer [83-85]. Although there is no similar studies conducted in leukemia patient, it is not a stretch to employ exosome-based immunotherapy alone or in combination with standard chemotherapy to treat leukemia, as illustrated in Figure 3. Another therapeutic approach which has attracted considerable attention involves the utility of exosomes as an efficient *in vivo* delivery system for drugs, proteins, miRNA, small interfering RNA (siRNA), antibodies, and many other molecules. With the aid of rapid progress in bioengineering field, we would anticipate a line of such exosome-wrapped novel therapeutic agents will be tested in clinical trials in near future. A deep understanding the exact characteristics, biogenesis and function of leukemia exosomes, as well as in the *in vivo* impact of exosomes on the different components of host immune system, may improve our ability to utilize exosomes as biomarkers, therapeutic vehicles and therapeutic targets.
